# Bone mesenchymal stem cells improve cholestatic liver fibrosis by targeting ULK1 to regulate autophagy through PI3K/AKT/mTOR pathway

**DOI:** 10.1093/stcltm/szae028

**Published:** 2024-05-13

**Authors:** Tingjuan Huang, Chunhong Zhang, Ziyi Shang, Qizhi Shuai, Lina Nie, Junjie Ren, Shulin Hou, Jun Xie

**Affiliations:** Key Laboratory of Birth Defect and Cell Regeneration, Department of Biochemistry and Molecular Biology, Shanxi Medical University, Taiyuan, 030001 Shanxi, China; Key Laboratory of Coal Environmental Pathogenicity and Prevention, Shanxi Medical University, Taiyuan, 030001 Shanxi, China; Key Laboratory of Birth Defect and Cell Regeneration, Department of Biochemistry and Molecular Biology, Shanxi Medical University, Taiyuan, 030001 Shanxi, China; Key Laboratory of Birth Defect and Cell Regeneration, Department of Biochemistry and Molecular Biology, Shanxi Medical University, Taiyuan, 030001 Shanxi, China; Key Laboratory of Birth Defect and Cell Regeneration, Department of Biochemistry and Molecular Biology, Shanxi Medical University, Taiyuan, 030001 Shanxi, China; Key Laboratory of Coal Environmental Pathogenicity and Prevention, Shanxi Medical University, Taiyuan, 030001 Shanxi, China; Key Laboratory of Birth Defect and Cell Regeneration, Department of Biochemistry and Molecular Biology, Shanxi Medical University, Taiyuan, 030001 Shanxi, China; Department of Gastroenterology and Hepatology, The First Hospital of Shanxi Medical University, Taiyuan, 030001 Shanxi, China; Key Laboratory of Birth Defect and Cell Regeneration, Department of Biochemistry and Molecular Biology, Shanxi Medical University, Taiyuan, 030001 Shanxi, China; Key Laboratory of Coal Environmental Pathogenicity and Prevention, Shanxi Medical University, Taiyuan, 030001 Shanxi, China; Key Laboratory of Birth Defect and Cell Regeneration, Department of Biochemistry and Molecular Biology, Shanxi Medical University, Taiyuan, 030001 Shanxi, China; Key Laboratory of Coal Environmental Pathogenicity and Prevention, Shanxi Medical University, Taiyuan, 030001 Shanxi, China

**Keywords:** cholestatic liver fibrosis, hepatic stellate cells, BMSCs, autophagy, ULK1, PI3K/AKT/mTOR

## Abstract

Cholestatic liver disease (CLD) is a severe disease, which can progress to liver cirrhosis, even liver cancer. Hepatic stellate cells (HSCs) activation plays a crucial role in CLD development. Bone mesenchymal stem cells (BMSCs) treatment was demonstrated to be beneficial in liver diseases. However, the therapeutic effect and mechanism of BMSCs on CLD are poorly known. In the present study, we investigated the therapeutic effects and underlying mechanisms of BMSCs transplantation in mouse models of bile duct ligation-induced cholestatic liver fibrosis (CLF). The results revealed that BMSCs significantly improved liver function and reduced the formation of fibrosis after portal vein transplantation. Mechanistically, after coculturing BMSCs and HSCs, we identified that BMSCs alleviated starvation-induced HSCs activation. Further, BMSCs inhibited HSCs activation by decreasing autophagy, and PI3K/AKT/mTOR pathway was involved in the regulation. More importantly, ULK1 is identified as the main autophagy-related gene regulated by BMSCs in HSCs autophagy. Overexpression of ULK1 reversed the suppression of HSCs autophagy by BMSCs. Collectively, our results provide a theoretical basis for BMSCs targeting ULK1 to attenuate HSCs autophagy and activation and suggest that BMSCs or ULK1 may be an alternative therapeutic approach/target for the treatment of CLF.

## Graphical Abstract

**Graphical Abstract:**
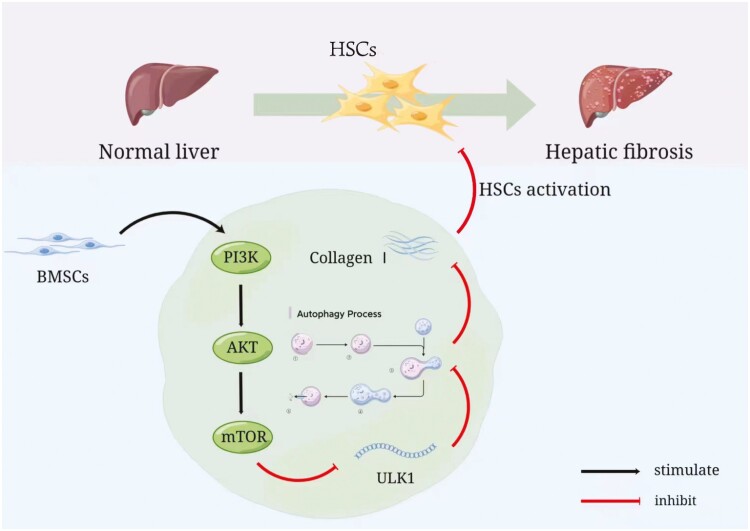

**Significance Statement**

This study represents that Bone mesenchymal stem cells or ULK1 may be a target for inhibiting HSCs activation, thus suppressing liver fibrosis, which provide potential therapeutic strategies for clinical cholestatic liver fibrosis.

## Introduction

Cholestatic liver disease (CLD) is due to cholestasis caused by gallstones, parasites, etc., resulting in the accumulation of bile acid in the liver and systemic circulation.^1^ During the occurrence and development of CLD, the gradual accumulation of bile acid leads to liver damage, inflammation, and fibrosis, and even develops into end-stage liver diseases such as cirrhosis and liver cancer.^2,3^ Globally, liver cirrhosis is still a major threat to health; there is a 74.53% increase in the number of liver cirrhosis patients from 1990 to 2017.^4^

At present, the clinical treatment of CLD can be carried out by removing the etiology and reducing cholestasis, but there is still a shortage of anti-hepatic fibrosis treatment. When the liver is damaged by the bile duct, quiescent hepatic stellate cells (HSCs) are activated and transdifferentiated into proliferative/activated HSCs,^5^ secreting excessive extracellular matrix. Due to the reversibility of liver fibrosis, therapeutic approaches based on delaying the fibrosis process by targeting HSCs are potentially effective intervention strategies.^6^ Cell therapy is currently a focus of clinical disease research. Based on the potential ability to repair or regenerate damaged tissues and to suppress immunological responses, mesenchymal stem cells (MSCs) are increasingly being applied as a treatment for liver diseases.^7,8^

MSCs can be isolated from a wide variety of sources, with bone mesenchymal stem cells (BMSCs) being the first to be discovered. Many investigations on the role of BMSCs in liver diseases have shown that BMSCs are effective. Previous studies have shown that BMSCs alleviated liver cirrhosis in vivo by downregulating ALT and AST and preventing HSCs activation in vitro.^9^ More interestingly, in vivo administration of human BMSCs in a rat liver fibrosis model induced by CCL4 effectively attenuated liver fibrosis, as indicated by a reduction of alpha-smooth muscle actin (α-SMA) expression as well as inflammation, thus restoring liver function.^10^ Similarly, mouse BMSCs efficiently exerted therapeutic activity in autoimmune hepatitis-related liver necrosis and inflammatory reaction.^11^ However, the therapeutic effect of BMSCs on CLD is poorly known, and the mechanism remains unclear.

Since autophagy has been reported to affect the activation of HSCs, it plays a crucial role in regulating liver fibrosis and its consequences.^12,13^ Autophagy is a lysosomal degradation pathway that occurs at basal levels in all cell types and is upregulated under stress conditions such as nutrient shortage and hypoxia.^14^ Autophagy serves an adaptive protective role, which is carried out by a molecular machinery encoded by autophagy-related genes (ATGs) and is pivotal to increase the cell survival by facilitating adaptive responses that protect against or lessen endoplasmic reticulum (ER) stress and mitochondrial dysfunction while maintaining proper metabolic energy and redox status.^15,16^ However, in certain diseases, the pro-survival functions of autophagy may be deleterious, as our previous research found that autophagy promoted activation of HSCs and exacerbated liver fibrosis.^17^

ATGs can be used as biomarkers for many diseases. A report showed that 2 autophagic markers, Beclin1 and LC3, were elevated in the serum of patients with acute ischemic stroke, which were correlated with good prognosis and low incidence of neurological dysfunction.^18^ Moreover, there was a significant relation between p62 concentrations in cerebrospinal fluid and clinical features of dementia patients, indicating that p62 could represent of a potential biomarker of neurodegeneration.^19^ Besides, the autophagy related gene markers including p62, LC3, Beclin1 also be relevant to the diabetic kidney diseases and acute respiratory distress syndrome.^20,21^ However, the lack of specific autophagy inhibitors targeting HSCs has become an obstacle to the therapy of cholestatic liver fibrosis (CLF). Therefore, it is necessary to explore the mechanism of autophagy in CLF and the analysis of autophagy biomarkers may be useful for the prediction and diagnosis of CLF.

Herein, we focused on the targeted ATG regulated by BMSCs, which additionally affect the autophagy and activation of HSCs. Our findings demonstrated that BMSCs exerted a therapeutic effect on CLF, providing a promising strategy for CLF. Furthermore, we observed that BMSCs inhibited autophagy in HSCs by targeting the ATG ULK1, ultimately reducing HSCs activation. Meanwhile, PI3K/AKT/mTOR signaling pathway participated in the modulation of ULK1 by BMSCs.

## Materials and methods

### Isolation, culture, and identification of BMSCs

BMSCs were isolated and extracted from the femur and tibia bone marrow of 3- to 4-weeks old SD rats, washed 3 times with phosphate-buffered saline (PBS; Procell), and then cultured in DMEM/F12 (Gibco) medium supplemented with 10% fetal bovine serum (FBS; Procell) at 37 °C in a 5% CO_2_ incubator. After 3 days, the floating cells were removed by changing the solution, and the remaining adherent cells were continued to be cultured in the original culture medium. When the attached BMSCs proliferated to more than 80% in T75 culture bottle, passage was carried out. Finally, P3 generation BMSCs were used for flow identification and subsequent experiments.

The P3 BMSCs were digested with 0.25% trypsin containing EDTA and divided into 3 tubes with 5-10 × 10^5^ cells per tube. Centrifuge each tube of cells at 350*g* for 5 minutes to remove the culture medium, followed by resuspended with 100 μL staining buffer and placed in flow cytometry tube. Subsequently, the antibodies against rat CD90, CD29, CD45, and CD11b were, respectively, added to 2 tubes based on fluorescence and incubated on ice in dark for 20 minutes. After washing twice with staining buffer, the phenotypes of BMSCs can be tested on the machine.

### Transplantation of BMSCs into CLF mice

Clean C56BL/6 wild male mice weighing about 25 g and aging about 8 weeks were provided by the Animal Center of Shanxi Medical University. All mice were raised in the animal room of Shanxi Medical University with 23 ± 2 °C room temperature and approximately 50% humidity. All mice were free diet. All animal experiments were reviewed and approved by the Laboratory Animal Ethics Committee of Shanxi Medical University.

According to our previous method,^17^ a CLF model was prepared by bile duct ligation (BDL), and a Sham group was established as a control (*n* = 10 mice/group). The mice in the BDL + BMSC group (*n* = 10) were injected with 1 × 10^6^ P3 BMSCs suspended in 100 µL of PBS into the liver through the portal vein before closing the abdomen after ligation of the common bile duct, and the abdominal cavity was closed after compression with a cotton swab for 1 minute to stop bleeding. The mice in BDL group only received 100 µL PBS. Each group of mice was sacrificed at 1, 2, and 4 weeks, and serum and liver tissue were obtained for subsequent testing.

### Coculture of BMSCs and HSCs

Mouse HSCs line JS-1 were cultured in RPMI 1640 medium (Gibco) containing 10% FBS, 100 U/mL penicillin, and 100 U/mL streptomycin. BMSCs and JS-1 were cocultured in Transwell cell culture chamber (Corning) with BMSCs in the lower layer and JS-1 in the upper layer. JS-1 cells were fused to 40%-50% in a T25 culture bottle, cultured with 2% FBS for 16 hours, and then transferred to the lower layer of the Transwell chamber on a 12-well plate. After 6-8 hours of adherent growth, the cells were transfected with ULK1 plasmid and siULK1 for 6 hours. Finally, the cells were cultured with 2% FBS and added diluted rapamycin (R8140, Solarbio) or 3-MA (GC10710, GLPBIO). At the same time, BMSCs were placed in the upper layer chamber. The cells were collected after cocultivation for 48 hours.

### Induction of osteogenesis and adipogenesis differentiation of BMSCs

Rat BMSCs Osteogenic Differentiation Kit (PD-008, Procell) was performed. When the fusion of BMSCs in the 6-well plate reached to 70%-80%, the culture medium was removed and 2 mL of osteogenesis differentiation medium was added to each well. The medium was changed every 3 days. After 2-4 weeks of induction, the osteogenic differentiation medium in the well plate was aspirated depending on the morphological changes and growth status of the cells, and rinsed with PBS 1-2 times. 10% neutral formaldehyde was added to fix for 30 minutes, formaldehyde removed, and rinsed with PBS 1-2 times. To each well 1 mL of alizarin red staining solution was added and stained at room temperature for 30 minutes. After cleaning the background impurities with PBS, the induction and staining effects could be observed under microscope.

Rat BMSCs Adipogenic Differentiation Kit (PD-008, Procell) was performed. The adipogenesis differentiation induction medium ADP1 and ADP2 were used for alternate induction. When significant and sufficient fat drops were observed in BMSCs, the cells were cultured with ADP2 for 3-6 days until the fat drops were large and full enough. The induction effect was determined by Oil Red O staining.

### ALT and AST detection

Alanine Aminotransferase (ALT, SEA207Mu, Cloud-Clone Corp Wuhan) and Aspartate Aminotransferase (AST, SEB214Mu, Cloud-Clone Corp Wuhan) were determined in mouse serum by ELISA.

### H&E staining

The fixed liver tissues by formaldehyde were embedded in paraffin and sliced into 4 µm. Subsequently, conventional xylene dewaxing was carried out and various levels of ethanol were hydrated. After staining with hematoxylin for 5 minutes, wash the slices with tap water, then differentiate with hydrochloric acid ethanol for 30 seconds, and finally stain with eosin for 2 minutes.

### Sirius red

The liver tissues were sliced into 6 μm and subjected to conventional dewaxing and hydration. Sirius red dye was dripped for 1 hour. Rinse slightly with running water to remove the stain on the surface of the slice. Mayer’s hematoxylin staining solution stained the nucleus for 8-10 min.

### Immunohistochemical Staining

As described previously, the formalin-fixed paraffin-embedded liver tissues were sliced into 3 μm. After sodium citrate repair treatment, the sections were combined with the primary antibody α-SMA (1:500, ab124964, Abcam), collagen I (1:300, ab270993, Abcam), and corresponding second antibody. Finally, DAB (ZSGB-BIO, Beijing) was used for color rendering.

### Cell proliferation

The 100 μL of cell suspension at a density of 2000 cells were seeded into per well of 96-well plate and incubated for 24, 48, and 72 hours respectively. Then, each well was added with 10 μL CCK8 solution and incubated for 4 hours. The absorbance was measured at 450 nm using an enzyme-linked immunosorbent assay.

### Wound scratch healing assay

The cells in each group were seeded at a concentration of 5 × 10^5^ cells per well in a 6-well plate. When the cells reached a convergence rate of about 80%, the 20 μL gun head was perpendicular to the surface of the plate and scratched from one end of the hole to the other end, and a small area was scratched. The cells were washed with sterile PBS for 3 times and then placed in incubator for 24 hours. The scratched area was imaged by a microscope.

### Western blot

The proteins were obtained by pre-cooled RIPA buffer (Beyotime, Shanghai, China) containing proteases and phosphatase inhibitors (Solarbio, Beijing, China). After quantification of protein concentration by BCA assay kit (Epizyme, Shanghai, China), 5× loading buffer was added to all samples and heated at 100 °C for 5 minutes. The protein expression was detected and analyzed by SDS-PAGE and immunoblotting. Primary antibodies in this study including α-SMA (1:1000, ab124964, Abcam), collagen I (1:1000, ab270993, Abcam), LC3 (1:1000, ab48394, Abcam), SQSTM1/p62 (1:1000, ab91526, Abcam), ULK1 (1:800, sc-390904, Santa), PI3K (1:1000, ab302985, Abcam), P-PI3K (1:1000, ab278545, Abcam), AKT (1:1000, ab8805, Abcam), P-AKT (1:1000, ab81283, Abcam), mTOR (1:1000, ab134903, Abcam), and P-mTOR (1:1000, ab109268, Abcam) were incubated at 4 °C overnight. Secondary antibodies were incubated at room temperature for 2 hours.

### RT-qPCR

Total RNAs were extracted using Trizol reagent (Invitrogen) and then converted to cDNA by using the PrimeScript RT reagent Kit with gDNA Eraser (RR047A, Takara). Real-time qPCR was performed by using the 2× TB Green Premix Ex Taq (RR820Q, Takara) in a 20-µL reaction system following the manufacturer’s guidelines. The CT values of target genes were normalized to that of the internal reference GAPDH to calculate the 2^–ΔΔCt^ value. The primers used in the study are listed in Table 1.

**Table 1.** Primer sequences for qRT-PCR.

Gene	Forward primer (5ʹ-3ʹ)	Reverse primer (5ʹ-3ʹ)
*α-SMA*	CTCCATCGTCCACCGCAAAT	GGCCAGGGCTACAAGTTAAGG
*Collagen I*	GCTCCTCTTAGGGGCCACT	ATTGGGGACCCTTAGGCCAT
*LC3B*	TGTGTCCACTCCCATCTCCGAAG	CCATTGCTGTCCCGAATGTCTCC
*SQSTM1/p62*	AGGAGGAGACGATGACTGGACAC	TTGGTCTGTAGGAGCCTGGTGAG
*ULK1*	TTCAGCACCAGCCGCATTACG	CAAAGCCAGCAGAGGGAGCAATC
*Beclin1*	GATCCACTGAGCACCGA	CTCACCTGGTGGCATTGTG
*Atg4b*	TGGAGTCAGAGAGGCACTGTAACG	TGTCTGTCAGTCCCAGGCGAAG
*Atg9a*	CAAGCCCGCCTCCAAGTACATG	TCCACAGCCAACACATCTTCATCG
*Atg12*	CGGACCATCCAAGGACTCATTGAC	TGGGGAAGGGGCAAAGGACTG
*Atg13*	ACCGATTGTCACTGCTGCTGAAG	TGCCCTTGCTTCCTGGAGAGTC
*Atg14*	AAGGAGAAGATTCAGCGGCACAAC	CATTGGGAAGATGACAGAGGTGAGC
*Atg16L1*	CAAGCCGAATCTGGACTGTGGATG	CGGTCGTGACTTCCTGAGACAATC
*PI3K*	TTGACAGTAGGAGGAGGTTGGA	TGCGTCAGCCACATCAAGTAT
*AKT*	GCTGGAGGACAACGACTACG	CTTCTCGTGGTCCTGGTTGTA
*mTOR*	TTCAGCCCTTCTTTGACAACA	CGATCATCTCGATTCATACCCT
*GAPDH*	AGTCTGTGTAGTTAGAAGCTCCA	TGGTCCAGGGTTTCTTACTCC

### Immunofluorescence Staining

The cells were cultured in Transwell chamber on a 12-well plate (Corning) and fixed with 4% paraformaldehyde (Coolaber) for 15 minutes, permeabilized with 0.5% Triton X-100 (Thermo Fisher) for 15 minutes, and then blocked with 5% BSA for 1 hour at room temperature. Then the cells were incubated with rabbit monoclonal α-SMA antibody (1:100) and LC3B (1:100) overnight at 4 °C followed by incubation with the fluorescent labeled secondary antibody for 1 hour at 37 °C. Finally, the cells were incubated with DAPI (Solarbio) in dark for 5 minutes for nuclear staining. Images were observed and collected under a fluorescence microscope (BX43, Olympus).

### Transmission electron microscopy

The cells to be assayed were washed twice with PBS and fixed in 2% glutaraldehyde for 2 hours, followed by 1% osmic acid for 2 hours. Samples were dehydrated increasing concentrations of ethanol and acetone, and saturated at room temperature in the mixtures of acetone and embedding medium for 2-4 hours. The samples were embedded with epoxy resin in 40 °C oven for 24 hours, and then polymerized for 48 hours at 60 °C. Ultrathin slices of 60 nm were stained with 1% uranium acetate and lead citrate for 15-30 minutes. Transmission electron microscopy was performed (JEOL, Japan).

### Cell apoptosis

Apoptosis was assessed using an Annexin V/PI apoptosis kit (SC123-02, Seven, Beijing, China) and flow cytometry (Beckman). 5 × 10^5^ cells of each group were collected, washed, and suspended in 100 µL binding buffer. Then, 2 µL Annexin V-FITC and 4 µL PI were added, followed by incubation at room temperature in dark for 15 minutes. Cell cycle distribution was determined using a flow cytometry according to the manufacturer’s instructions.

### Statistical analysis

All data were expressed as mean ± standard error of the mean (SEM), and all calculations were performed using GraphPad Prism 8.0 or SPSS 23.0 statistical software. In addition, statistical significance was evaluated using Student’s t test or one-way analysis of variance. *P*-value of <0.05 was considered statistically significant.

## Results

### BMSCs infusion improved liver function and inflammatory response of BDL mice

**Figure 1.**
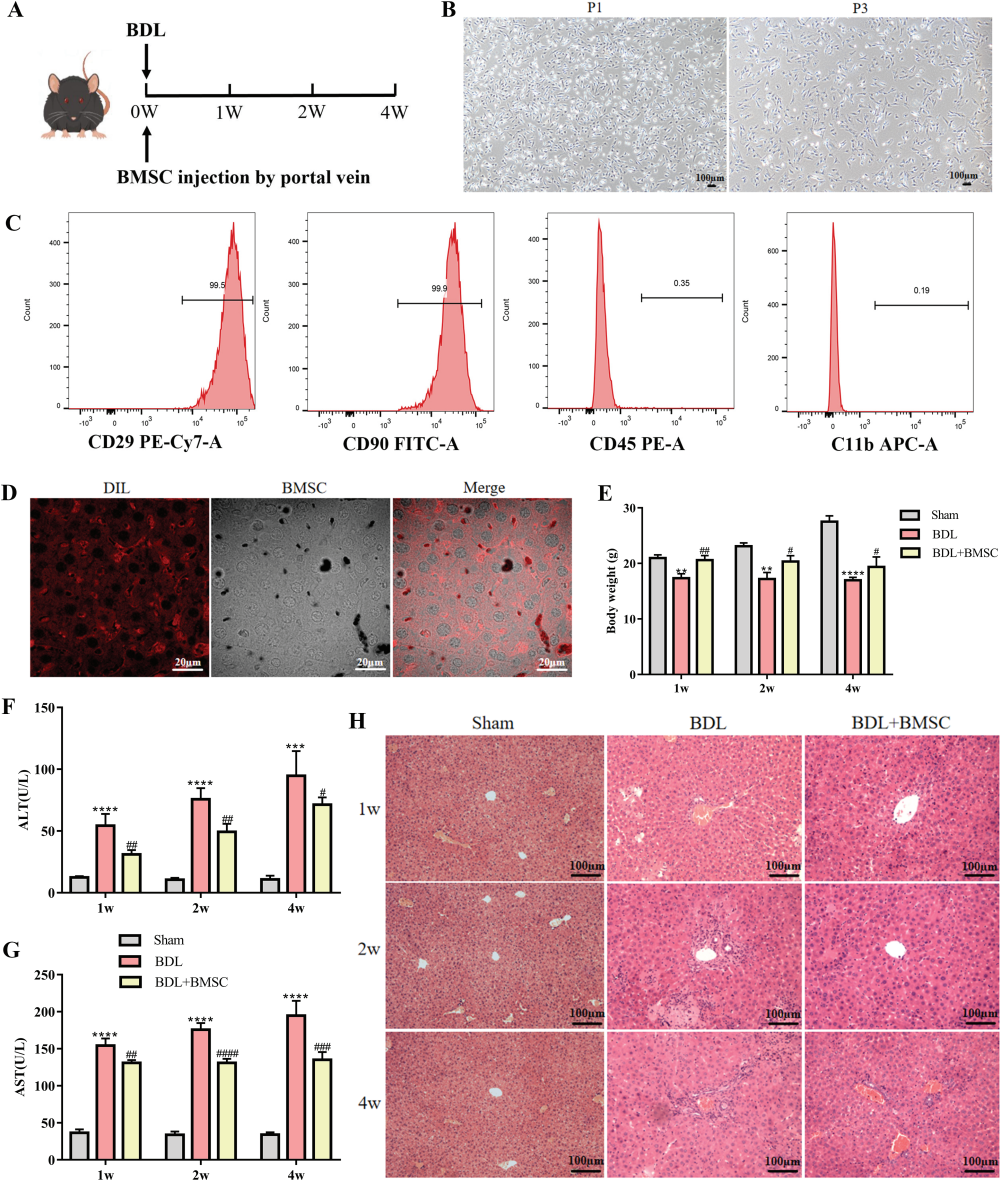
BMSCs infusion improved liver function and inflammatory response of BDL mice. (A) Schematic representation of the timing strategy used to evaluate the role of BMSCs transplantation in BDL-induced liver fibrosis. (B) Rat bone marrow-derived MSCs showed spindle cell morphology. Scale bar = 100 µm. (C) Evaluation of BMSCs markers by flow cytometry; *n* = 3 independent experiments. (D) DIL staining was performed to assess the localization of BMSCs in the liver on day 1; *n* = 8 animals. Scale bar = 20 µm. (E) Body weight of mice in BDL-induced mice liver after BMSCs transplantation; *n* = 8 animals. (F, G) Determination of serum concentration of ALT and AST in BDL-induced mice liver after BMSCs transplantation; n = 8 animals. (H) Representative liver stained images by HE and Sirius red staining in BDL-induced mice liver after BMSCs injected; *n* = 8 animals. Scale bar = 100 µm. All data are presented as mean ± SD. **P < .01, ***P < .001, ****P < .0001 vs sham. ^##^*P* < .01, ^###^*P* < .001, ^####^*P* < .0001 vs BDL.

In this study, we investigated whether BMSCs from rats have a therapeutic effect on CLF. The transplantation strategy of BMSCs is illustrated in Figure 1A. We first identified the morphology and phenotypes of BMSCs. Our data showed that both the primary and P3 BMSCs appeared as spindle-shaped cells (Figure 1B). The results from flow cytometry displayed the positive expression of CD29 and CD90 and the negative expression of CD45 and CD11b, which indicated that the extracted cells were BMSCs (Figure 1C). The localization of transplanted BMSCs in the liver is vital for elucidating their functions. DIL (1,1ʹ-dioctadecyl-3,3,3ʹ,3ʹ-tetramethylindocarbocyanine perchlorate), the most commonly used cell membrane fluorescent probes, is a lipophilic membrane dye that can stain the entire cell membrane upon entering the cell membrane. We followed the distribution of BMSCs labeled with DIL in the liver after injection into the mouse through portal vein. One day later, we observed a significant distribution of BMSCs in the liver (Figure 1D). Meanwhile, compared to the BDL group, mice treated with BMSCs all showed significant weight gain at 1, 2, and 4 weeks (Figure 1E).

Next, we examined the protective effect of BMSCs on hepatocyte function. The results showed that BMSCs significantly reduced the increases in the levels of ALT and AST in the serum of cholestatic mice at 1, 2, and 4 weeks (Figure 1F, G). Furthermore, the inflammatory cell infiltration was slightly lower in the livers of BMSCs-transplanted mice than in those of non-BMSCs-treated BDL mice at 3 time points (Figure 1H). These results indicated that BMSCs transplantation improved liver function and liver cell inflammation caused by cholestasis.

### BMSCs transplantation attenuated CLF

**Figure 2.**
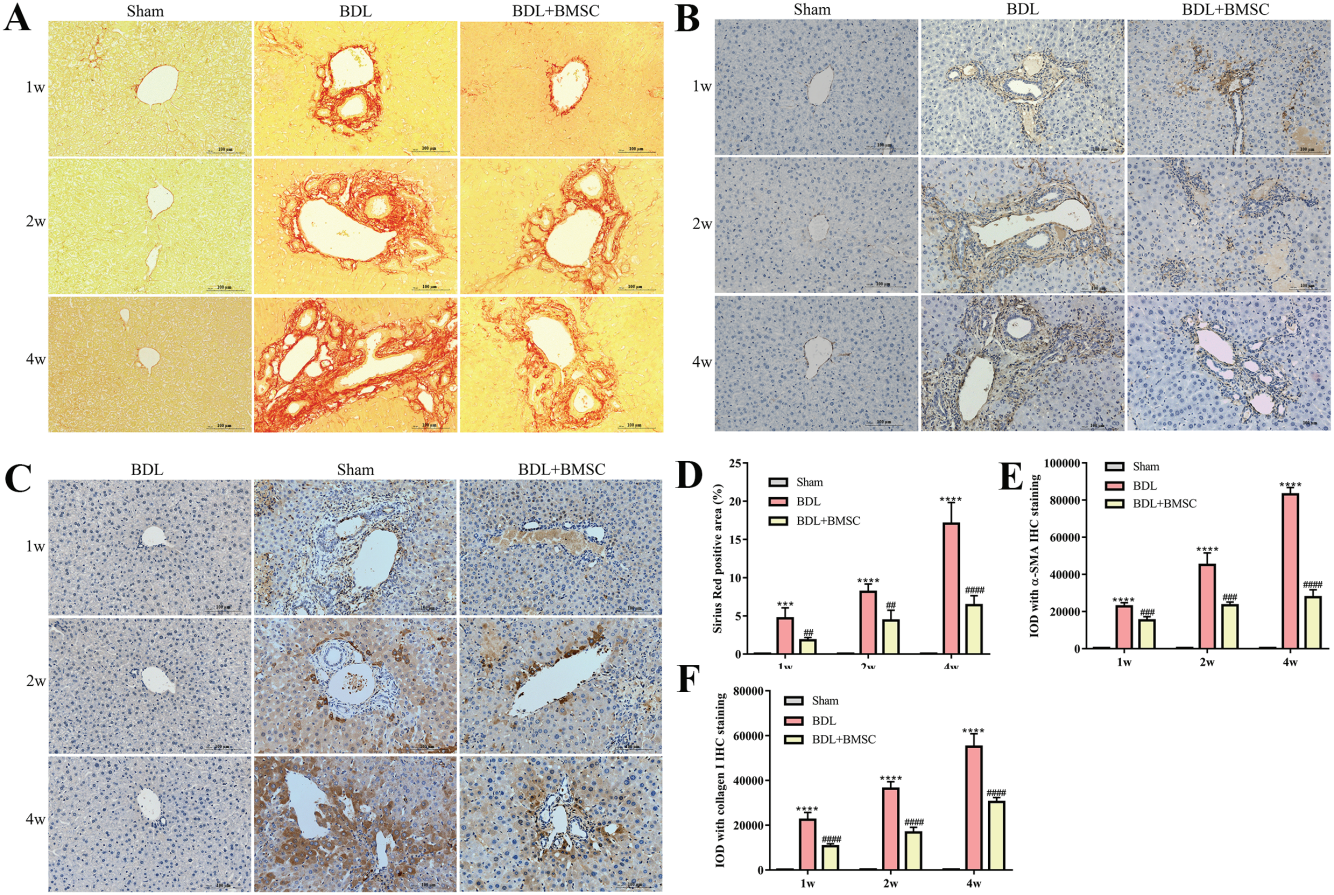
BMSCs transplantation attenuated cholestatic liver fibrosis. (A, D) Representative liver stained images by sirius red staining in BDL-induced mice liver after BMSCs were injected; *n* = 8 animals. Scale bar = 100 µm. (B, C, E, F) α-SMA and collagen I were semi-quantitatively detected by immunohistochemical staining; *n* = 8 animals. Scale bars = 100 µm. All data are presented as mean ± SD. **P < .01, ***P < .001, ****P < .0001 vs sham. ^##^*P* < .01, ^###^*P* < .001, ^####^*P* < .0001 vs BDL.

Then, we further detected the therapeutic effect of BMSCs on CLF in mice. Sirius red results showed a significant reduction in the production of collagen fibers in BMSCs-treated mice at 1, 2, and 4 weeks compared with BDL group (Figure 2A, D). Accordingly, consistent with the prediction, the expression of α-SMA and collagen I was markedly blocked in BMSCs recipients compared to non-BMSCs-treated BDL mice at 3 time points (Figure 2B, C, E, F). These data show that BMSCs transplantation alleviated collagen fiber formation caused by cholestasis.

### BMSCs reduced autophagy and inhibited activation in HSCs

**Figure 3.**
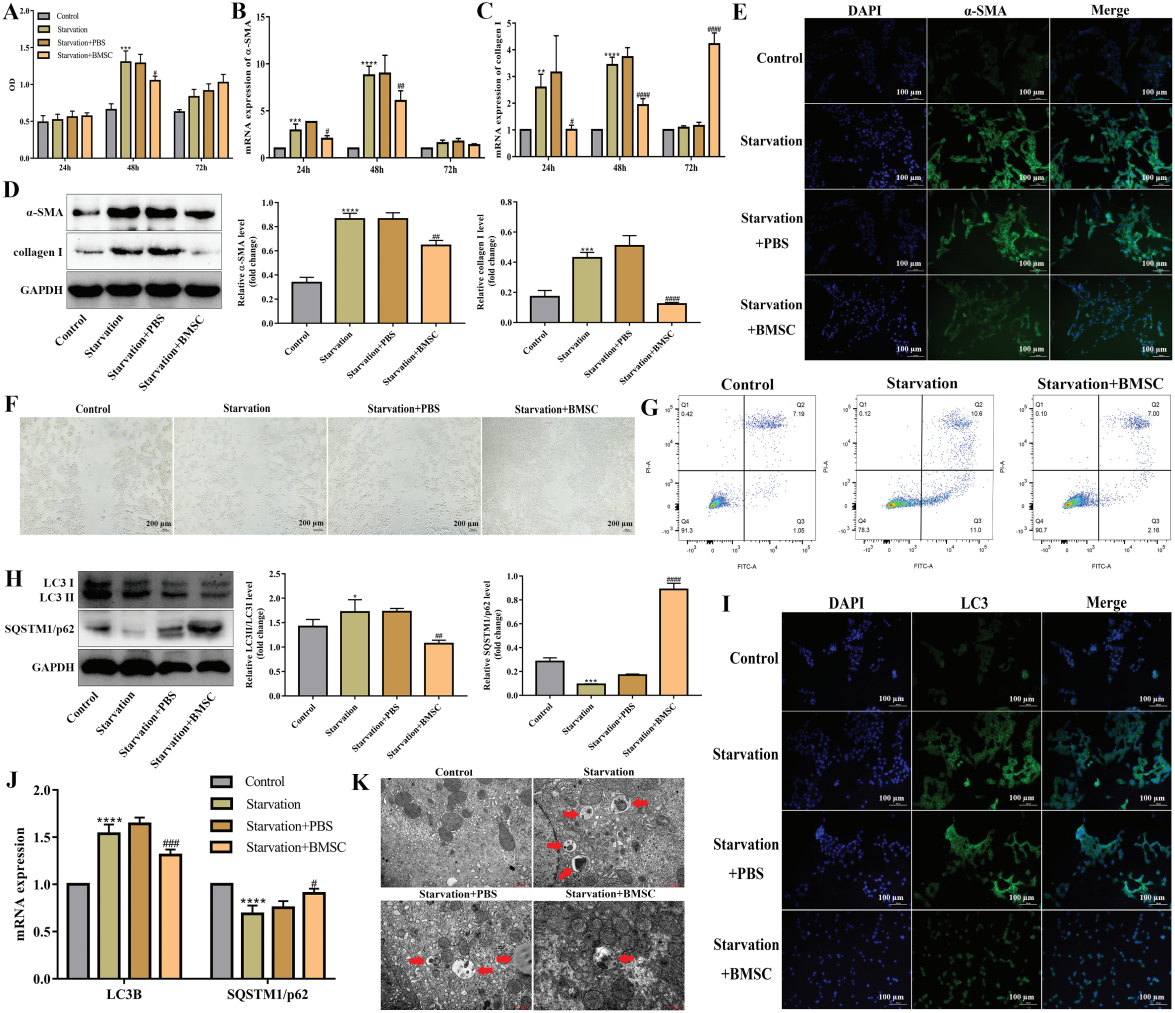
BMSCs reduced autophagy and inhibited activation in HSCs***. (A) Cell proliferation in starvation-induced JS-1 cells was detected using CCK8 after coculture with BMSCs at 24, 48, and 72 hours time points; *n* = 3 independent experiments. (B, C) mRNA levels of α-SMA and collagen I with qPCR in starvation-induced JS-1 cells cocultured with BMSCs at 24, 48, and 72 hours time points; *n* = 3 independent experiments. (D, H) Protein levels of α-SMA, collagen I, LC3 II/LC3 I, and SQSTM1/p62 with western blot in starvation-induced JS-1 cells cocultured with BMSCs; *n* = 3 independent experiments. (E, I) Immunofluorescence of α-SMA and LC3 in starvation-induced JS-1 cells cocultured with BMSCs. The cells were stained for α-SMA and LC3B. Scale bars = 100 µm. (F) Migration in starvation-induced JS-1 cells was detected using scratch experiments after coculture with BMSCs; *n* = 3 independent experiments. Scale bars = 200 µm. (G) Apoptosis in starvation-induced JS-1 cells was analyzed using flow cytometry after coculture with BMSCs; *n* = 3 independent experiments. The Q1 quadrant represents necrotic cells, Q2 quadrant represents late apoptotic cells, Q3 quadrant represents early apoptotic cells, and Q4 quadrant represents living cells. (J) mRNA levels of LC3 and SQSTM1/p62 with qPCR in starvation-induced JS-1 cells cocultured with BMSCs; *n* = 3 independent experiments. (K) Transmission electron microscopy was used to assess autophagosome in starvation-induced JS-1 cells cocultured with BMSCs; *n* = 3 independent experiments. Scale bars = 1 µm. All data are presented as mean ± SD. *P < .05, **P < .01, ***P < .001, ****P < .0001 vs control, ^#^*P* < .05, ^##^*P* < .01, ^###^*P* < .001, ^####^*P* < .0001 vs starvation + PBS.

HSCs activation is a central link in the occurrence and development of liver fibrosis. To further investigate the effect of BMSCs transplantation on HSCs activation, we used starvation-induced mouse JS-1 cells as HSCs activation models. After coculturing with BMSCs for 24, 48, and 72 hours, the proliferation ability of JS-1 cells was detected. The results showed that BMSCs significantly inhibited HSCs activation after cocultivation for 48 hours (Figure 3A), and the mRNA-level detection of α-SMA and collagen I also displayed similar results (Figure 3B, C). Additionally, the western blot results revealed that α-SMA and collagen I expression was decreased in BMSCs coculture cells compared to non-coculture cells (Figure 3D), consistent with the immunofluorescence detection results of α-SMA (Figure 3E). Moreover, the results of cell migration ability by cell scratch experiment showed that compared to the PBS group, BMSCs dramatically increased cell migration ability (Figure 3F). Therefore, we demonstrated in vitro that BMSCs diminished HSCs activation.

Autophagy is critically involved in HSCs activation. To elucidate whether BMSCs have an impact on autophagy, we evaluated the levels of autophagy in HSCs. First, cell apoptosis showed that BMSCs reduced apoptosis in HSCs (Figure 3G). Next, western blot displayed that LC3 II/LC3 I was significantly reduced in the BMSCs-treated group compared with the PBS group, while SQSTM1/p62 showed the opposite trend (Figure 3H). Besides, immunofluorescence results showed that BMSCs reduced the expression of LC3 (Figure 3I). Consistent with these results, BMSCs decreased the mRNA level of LC3 and increased the SQSTM1/p62 (Figure 3J). Finally, we observed the effect of BMSCs on the number of HSCs autophagosomes through TEM, and the results revealed that BMSCs reduced the number of autophagosomes induced by starvation (Figure 3K). These data suggest that BMSCs could reduce HSCs autophagy.

### BMSCs suppressed HSCs activation through autophagy

**Figure 4.**
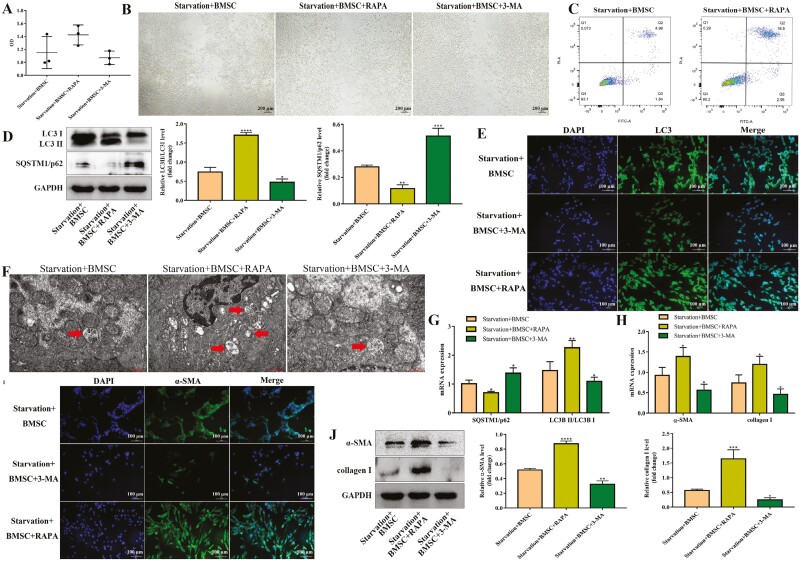
BMSCs suppressed HSCs activation through autophagy. (A) Starvation-induced JS-1 cells treated with RAPA or 3-MA, and then were cocultured with BMSCs. Cell proliferation in each group of JS-1 cells was detected using CCK8; *n* = 3 independent experiments. (B) Migration in each group of JS-1 cells was detected using scratch experiments; *n* = 3 independent experiments. (C) Apoptosis in each group of JS-1 cells was analyzed using flow cytometry; *n* = 3 independent experiments. The Q1 quadrant represents necrotic cells, Q2 quadrant represents late apoptotic cells, Q3 quadrant represents early apoptotic cells, and Q4 quadrant represents living cells. (D, J) Protein levels of α-SMA, collagen I, LC3 II/LC3 I, and SQSTM1/p62 with western blot in each group of JS-1 cells; *n* = 3 independent experiments. (E, I) Immunofluorescence of α-SMA and LC3 in each group of JS-1 cells. The cells were stained for α-SMA and LC3B. Scale bars = 100 µm. (F) Transmission electron microscopy was used to assess autophagosome in each group of JS-1 cells; *n* = 3 independent experiments. (G, H) mRNA levels of LC3 and SQSTM1/p62 with qPCR in each group of JS-1 cells; *n* = 3 independent experiments. Scale bars = 1 µm. All data are presented as mean ± SD. *P < .05, **P < .01, ***P < .001, ****P < .0001 vs starvation + BMSCs.

To verify the role of autophagy during HSCs activation regulated by BMSCs, we added autophagy inhibitor 3-MA and autophagy inducer RAPA in HSCs on the basis of BMSCs treatment. The results revealed that RAPA promoted the HSCs proliferation weakened by BMSCs, while 3-MA further inhibited it (Figure 4A). Furthermore, RAPA facilitated the migration of HSCs and 3-MA diminished it (Figure 4B). Cell apoptosis showed that RAPA increased apoptosis in HSCs reduced by BMSCs (Figure 4C). To clarify whether RAPA and 3-MA exerted regulation on autophagy, LC3 and SQSTM1/p62 were detected. As shown in Figure 4D, RAPA markedly increased the ratio of LC3 II/LC3 I weakened by BMSCs and decreased p62 expression while 3-MA reversed this change, consistent with RT-qPCR results (Figure 4G). Meanwhile, immunofluorescence suggested that the level of LC3 was increased by RAPA and decreased by 3-MA (Figure 4E). In addition, TEM confirmed that RAPA increased the number of autophagic lysosomes while 3-MA decreased it (Figure 4F). These results indicated that RAPA encouraged autophagy, while 3-MA developed the opposite effect.

Subsequently, the HSCs activation markers were further detected. The results suggested that the inhibition effect of BMSCs on α-SMA and collagen I was further strengthened by RAPA and weakened by 3-MA (Figure 4H, J). Immunofluorescence of α-SMA showed the same result (Figure 4I). All these findings supported that autophagy was required for the inhibitory effect of BMSCs on HSCs activation.

### PI3K/AKT/mTOR pathway was involved in the regulation of HSCs autophagy by BMSCs

**Figure 5.**
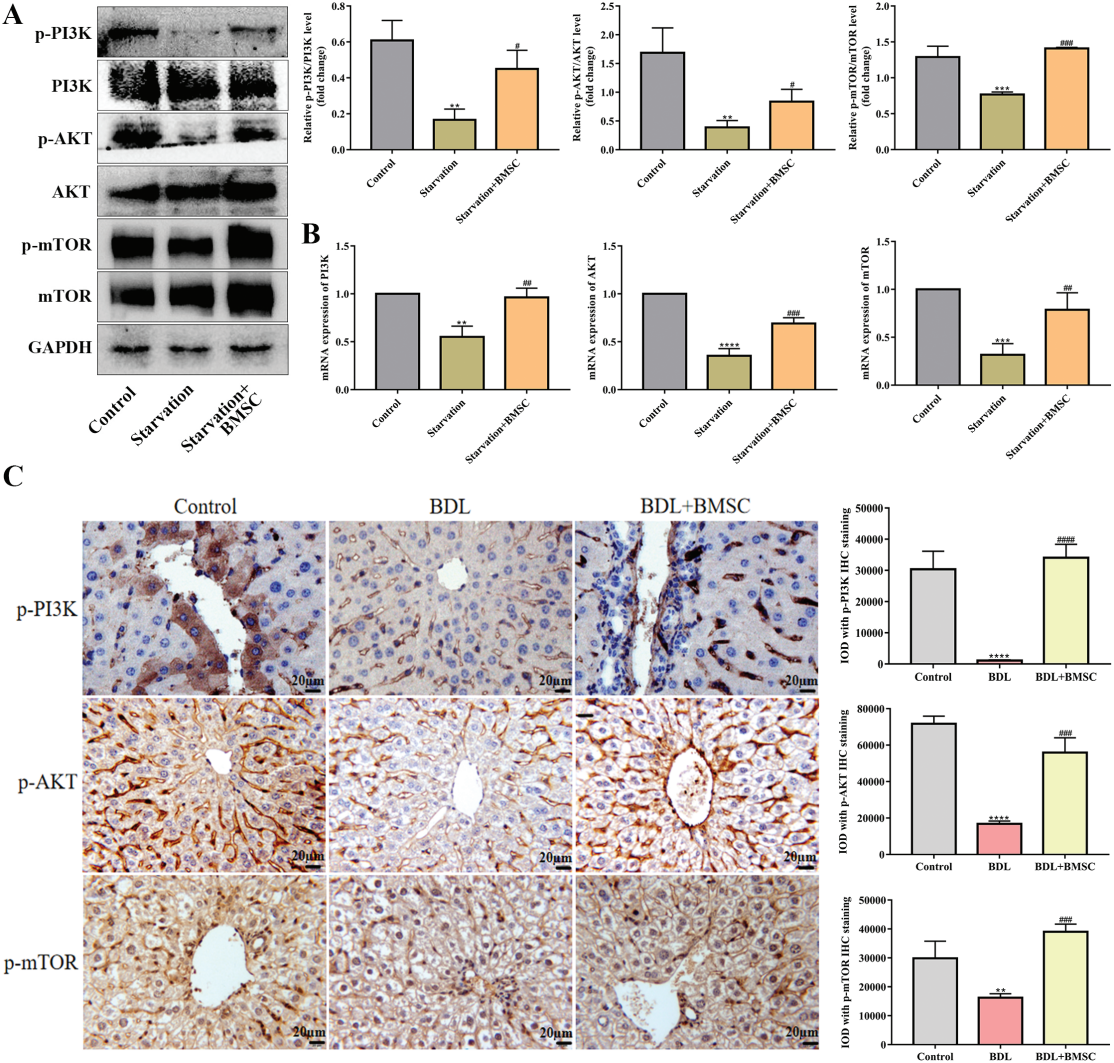
PI3K/AKT/mTOR pathway was involved in the regulation of HSCs autophagy by BMSCs. (A) Starvation-induced JS-1 cells were treated with RAPA or 3-MA, and then were cocultured with BMSCs. Protein levels of p-PI3K, PI3K, p-AKT, AKT, p-mTOR, and mTOR with western blot in each group of JS-1 cells; *n* = 3 independent experiments. (B) mRNA levels of PI3K, AKT, and mTOR with qPCR in each group of JS-1 cells; *n* = 3 independent experiments. (C) p-PI3K, p-AKT, and p-mTOR were semi-quantitatively detected by immunohistochemical staining in Control, BDL and BDL + BMSC group; *n* = 8 animals. Scale bars = 20 µm. All data are presented as mean ± SD. **P < .01, ***P < .001, ****P < .0001 vs control, ^#^*P* < .05 ^##^*P* < .01, ^###^*P* < .001 vs starvation or BDL.

Autophagy is a complicated process that is regulated by various signaling pathways, such as PI3K/AKT/mTOR.^22^ To clarify whether BMSCs inhibited autophagy in HSCs by activating the PI3K/AKT/mTOR pathway, we first measured the expression of total and phosphorylated PI3K, AKT, and mTOR. The western blot results showed that significant increases in the levels of p-PI3K, p-AKT, and p-mTOR in BMSCs-treated JS-1 cells compared with the starvation group (Figure 5A). In addition, the mRNA levels of PI3K, AKT, and mTOR were remarkably enhanced by treatment with BMSCs (Figure 5B), indicating that BMSCs inhibited HSCs autophagy and activation by upregulating PI3K/AKT/mTOR. Next, in order to verify that BMSCs eased liver fibrosis through PI3K/AKT/mTOR pathway, we detected its expression in vivo. Compared with the BDL group, the number of yellow puncta in BMSCs injection group was obviously increased (Figure 5C). Therefore, we concluded that PI3K/AKT/mTOR pathway profoundly participated in BMSCs-induced autophagy inhibition in HSCs.

### ULK1 is necessary for the protective roles of BMSCs in HSCs autophagy and activation

**Figure 6.**
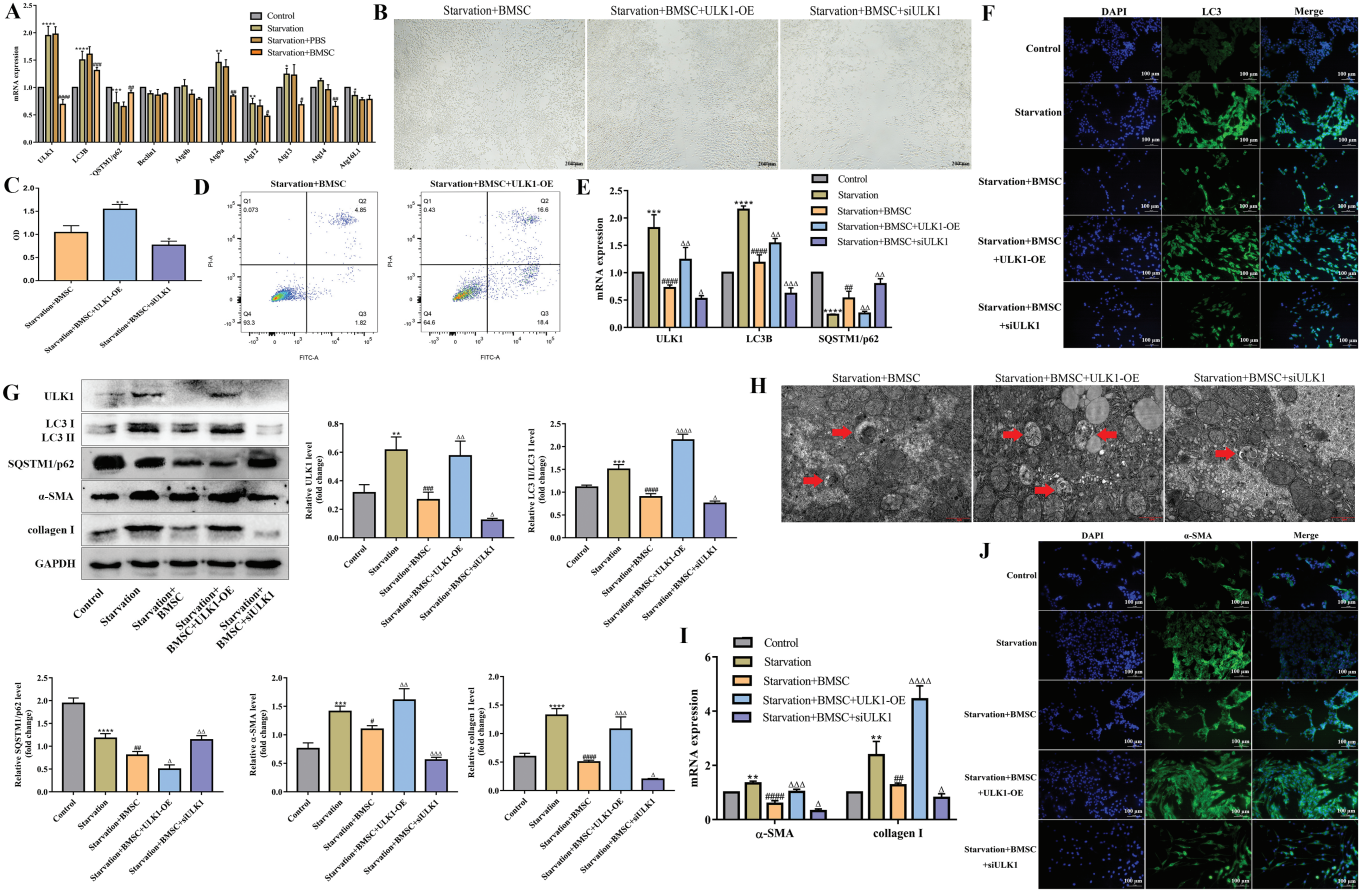
ULK1 is necessary for the protective roles of BMSCs in HSCs autophagy and activation. (A) mRNA levels of ULK1, LC3B, SQSTM1/p62, Beclin1, Atg4b, Atg9a, Atg12, Atg13, Atg14, and Atg16L1 were measured with qPCR in starvation-induced JS-1 cells cocultured with BMSCs; *n* = 3 independent experiments. (B) Starvation-induced JS-1 cells treated with ULK1-OE or siULK1, and then were cocultured with BMSCs. Migration in each group of JS-1 cells was detected using scratch experiments; *n* = 3 independent experiments. (C) Cell proliferation in each group of JS-1 cells was detected using CCK8; *n* = 3 independent experiments. (D) Apoptosis in each group of JS-1 cells was analyzed using flow cytometry; *n* = 3 independent experiments. The Q1 quadrant represents necrotic cells, Q2 quadrant represents late apoptotic cells, Q3 quadrant represents early apoptotic cells, and Q4 quadrant represents living cells. (E, I) mRNA levels of ULK1, LC3B, SQSTM1/p62, α-SMA, and collagen I were measured with qPCR in each group of JS-1 cells; *n* = 3 independent experiments. (F, J) Immunofluorescence of LC3 and α-SMA in each group of JS-1 cells. The cells were stained for LC3B and α-SMA. Scale bars = 100 µm. (G) Protein levels of ULK1, LC3 II/LC3 I, SQSTM1/p62, α-SMA, and collagen I were measured with western blot in each group of JS-1 cells; *n* = 3 independent experiments. (H) Transmission electron microscopy was used to assess autophagosome in each group of JS-1 cells; *n* = 3 independent experiments. Scale bars = 1 µm. All data are presented as mean ± SD. *P < .05, **P < .01, ***P < .001, ****P < .0001 vs control. ^#^*P* < .05 ^##^*P* < .01, ^###^*P* < .001, ^####^*P* < .0001 vs starvation. ^Δ^*P* < .05 ^ΔΔ^*P* < .01, ^ΔΔΔ^*P* < .001, ^ΔΔΔΔ^*P* < .0001 vs starvation + BMSCs.

To assess the targeted ATGs involved in the blocking effect of BMSCs on HSCs, we performed mRNA expression analysis on the main ATGs. The results showed that the ULK1 was the highest expression ATG in starvation-induced HSCs, which could be dramatically downregulated by BMSCs, suggesting that ULK1 is a key factor in BMSCs function (Figure 6A).

To further validate the function of ULK1 in BMSCs treatment of HSCs activation, we used plasmids to overexpress ULK1 and small interfering RNA to knockdown ULK1 in HSCs to perform rescue experiment. siULK1 obviously reduced the migration ability of HSCs increased by BMSCs (Figure 6B). Meanwhile, ULK1 overexpression increased cell proliferation, while ULK1 knockdown exerted the opposite effect (Figure 6C). Furthermore, ULK1 increased the number of apoptotic cells (Figure 6D). RT-qPCR revealed that ULK1-OE aggravated LC3 mRNA reduced by BMSCs and weakened SQSTM1/p62 enhanced by BMSCs while siULK1 played the opposite role (Figure 6E). Similar to RT-qPCR, immunofluorescence showed the same outcome (Figure 6F). In contrast, overexpression of ULK1 significantly increased while silencing ULK1 further sunk LC3 protein expression attenuated by BMSCs, the result of SQSTM1/p62 was opposite (Figure 6G). As expected, the effect of ULK1 on autophagosomes also showed the same results (Figure 6H).

Our studies exhibited that ULK1 participated in the regulation of HSCs autophagy by BMSCs. Next, we investigated whether ULK1 functioned in the inhibitory effect of BSMCs on HSCs activation. As shown in Figure 6G, the protein expression of α-SMA and collagen I decreased by BMSCs was increased upon ULK1 overexpression, while siULK1 had the opposite effect. In consistent, RT-qPCR and immunofluorescence displayed the same results (Figure 6I, J). These data indicated that ULK1 played an essential role in BMSCs-attenuated HSCs autophagy and activation.

### BMSCs targeted ULK1 through PI3K/AKT/mTOR pathway, thereby suppressing HSCs autophagy and activation

**Figure 7.**
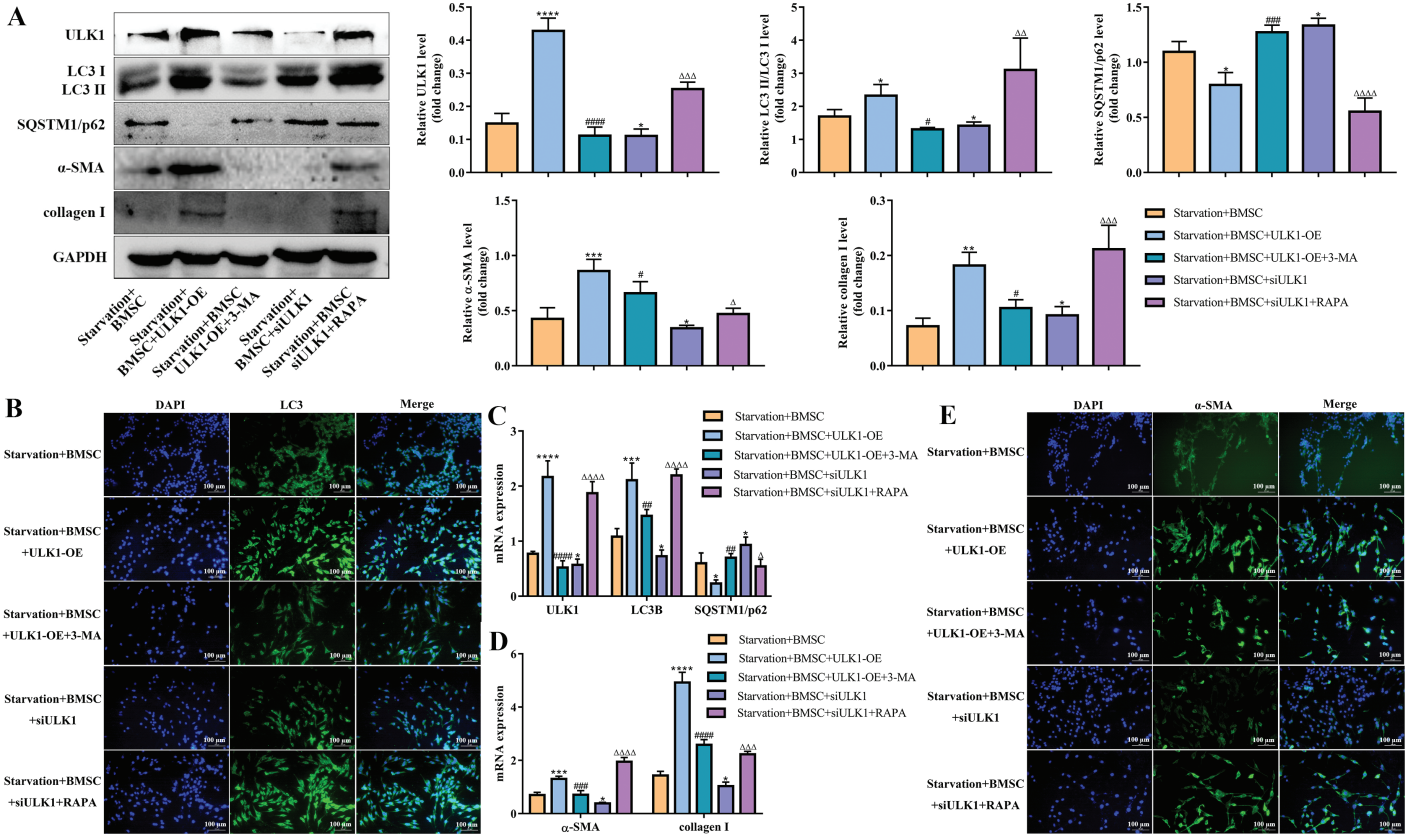
BMSCs targeted ULK1 through PI3K/AKT/mTOR pathway, thereby suppressing HSCs autophagy and activation. (A) Starvation-induced JS-1 cells were treated with ULK1-OE and 3-MA or siULK1 and RAPA, and then were cocultured with BMSCs. Protein levels of ULK1, LC3 II/LC3 I, SQSTM1/p62, α-SMA, and collagen I are analyzed using western blot in each group of JS-1 cells; *n* = 3 independent experiments. (B, E) Immunofluorescence of LC3 and α-SMA in each group of JS-1 cells. The cells were stained for LC3B and α-SMA. Scale bars = 100 µm. (C, D) mRNA levels of ULK1, LC3B, SQSTM1/p62, α-SMA, and collagen I with qPCR in each group of JS-1 cells; *n* = 3 independent experiments. All data are presented as mean ± SD. *P < .05, **P < .01, ***P < .001, ****P < .0001 vs starvation + BMSCs. ^#^*P* < .05 ^##^*P* < .01, ^###^*P* < .001, ^####^*P* < .0001 vs starvation + BMSC + ULK1-OE. ^Δ^*P* < .05 ^ΔΔ^*P* < .01, ^ΔΔΔ^*P* < .001, ^ΔΔΔΔ^*P* < .0001 vs starvation + BMSC + siULK1.

We have shown in earlier results that ULK1 is an efficient autophagy inhibitor in starvation-induced HSCs activation. ULK1 also exhibited autophagy-inhibiting properties even in the presence of BMSCs-treated. mTOR is a master regulator of autophagy and its activation leads to the suppression of autophagy. The activity of ULK1 is regulated by phosphorylation, in which mTOR-mediated phosphorylation inhibits ULK1 activity.^23,24^ To further excavate the role of PI3K/AKT/mTOR pathway on ULK1 in BMSCs-treated HSCs, we performed western blot analysis to assess the effects of PI3K/AKT/mTOR intervention on ULK1 and HSCs autophagy and activation. The ULK1 protein and mRNA were significantly downregulated by 3-MA and upregulated by RAPA (Figure 7A, C).

Subsequently, we explored HSCs autophagy and activation after intervention with PI3K/AKT/mTOR pathway on the basis of ULK1-ovexpression or knockdown. The western blot results indicated that 3-MA reversed the ratio of LC3 II/LC3 I increased by ULK1-OE in BMSCs-treated HSCs, while SQSTM1/p62 showed the opposite trend (Figure 7A). Meanwhile, RAPA promoted the ratio of LC3 II/LC3 I caused by ULK1 knockdown but inhibited the expression of SQSTM1/p62 (Figure 7A). Similar to the data in western blot, immunofluorescence and RT-qPCR showed the same results (Figure 7B, C). As expected, 3-MA reduced the protein and mRNA levels of α-SMA and collagen I increased by ULK1 overexpression and RAPA facilitated their expression restrained by ULK1 knock down in BMSCs-treated HSCs (Figure 7A, D, E), indicating that BMSCs regulated ULK1 through PI3K/AKT/mTOR pathway, thereby reducing autophagy and ultimately suppressing HSCs activation.

## Discussion

Mesenchymal stem cells, with their differentiation into hepatocytes,^25^ have broad ranging anti-inflammatory^26^ and immunomodulatory properties.^27^ They have the potential as a cellular therapy avenue for liver diseases. Several preclinical and clinical studies have focused on the beneficial therapeutic effects of MSCs in various liver diseases.^28-30^ Accumulating evidence showed that MSCs from different sources have protective effects on schistosome-infected, CCl4-induced, alcoholic, and non-alcoholic fatty liver diseases.^31-34^ In the present study, we used BMSCs as a cell source to investigate their beneficial effects on BDL-induced liver fibrosis and HSCs activation.

In 2006, the International Society for Cell Therapy established a set of minimal characteristics to define MSCs.^35^ According to literature reports (1994-2021), the BMSCs express cell surface markers CD90, CD105, CD29 but absence CD45, CD11b.^36^ Our result indicated that BMSCs from rat were successfully extracted. Moreover, MSCs can migrate to various damaged tissues, including the lung, liver, and spleen, along with the circulartion.^37^ Here, we found that BMSCs were implanted in the liver after portal vein transplantation. Increasing evidence has shown that MSCs treatment alleviated various liver diseases through anti-inflammatory regulation.^38,39^ For example, MSCs from mice increased IL-6-dependent hepatocyte proliferation and reduced inflammatory TNF-α cytokine secretion.^40^ Our data indicated that BMSCs improved liver function and exerted anti-fibrosis effects in the BDL mice by alleviating inflammatory injury, reducing collagen fiber generation, and promoting tissue repair, which is similar to the findings of Li et al.^41^ This is a novel finding that may be valuable for the treatment of CLD.

Activated HSCs are the major players in liver fibrosis. When cocultured with human embryonic stem cells (hE-MSCs), α-SMA expression in LX2 by TGFβ1 was suppressed.^42^ In our study, we initially transplanted BMSCs to recipient mice after ligation of the common bile duct. As we expected, BMSCs reduced the expression of α-SMA and collagen I, indicating that BMSCs attenuated CLF, consistent with this report.^43^

Our previous reports showed that autophagy contributed to HSCs activation.^17,44^ Additionally, research showed that human umbilical cord MSCs (huc-MSCs) treatment significantly improved pulmonary fibrosis by downregulating autophagy.^45^ Like this report, our results indicated that autophagy level along with HSCs activation were decreased by BMSCs. Autophagy agonist/inhibitor rapamycin/3-MA participates in the modulation of autophagy.^46^ In the present study, we focused on intervention in HSCs autophagy and found that BMSCs dramatically suppressed the activation of HSCs by reducing autophagy. Nevertheless, autophagy occurred in BMSCs during their application, especially in those exposed to stress conditions, and regulation of autophagy in BMSCs would be a promising strategy to improve the therapeutic efficacy.^47^ Therefore, it is necessary to further study the inhibitory effect of increased or decreased autophagy in BMSCs on HSCs activation.

As the most classic autophagy signaling pathway, the mTOR pathway, has been implicated as a critical regulator of autophagy, pharmacological targeting of mTOR activity has been an attractive therapeutic strategy.^48^ Consistent with our previous study,^17,44^ direct mTOR inhibitors, such as rapamycin, have been shown to promote autophagy and reduce apoptosis, thus stimulating HSCs activation, thereby promoting liver fibrosis.^49,50^ Here, we identified the downstream signaling pathway PI3K/AKT/mTOR by which BMSCs regulate HSCs autophagy. As expected, our results showed that BMSCs activated PI3K/AKT/mTOR pathway to inhibit the HSCs autophagy and activation.

Research has shown that ULK1 initiated the formation of autophagosome.^51^ Meanwhile, silencing ULK1 significantly blocked the autophagic flux.^52^ It is worth noting that our study found that compared to other ATGs, ULK1 is the main participant in BMSCs regulation of HSCs autophagy. ULK1 exerted function of promoting HSCs autophagy and activation inhibited by BMSCs. This is a novel mechanism for the activation of HSCs as it shows that serum levels of ULK1 may be used as a biomarker for diagnosis or treatment of CLF. Furthermore, the data revealed that ULK1 is a downstream regulatory protein of the PI3K/AKT/mTOR pathway. This further explains that ULK1 may be a potential target for regulating HSCs activation, which needs to be examined in blood and tissue samples of patients with CLF subsequently.

## Conclusions

In summary, our study showed that BMSCs inhibit HSCs activation by suppressing HSCs autophagy by targeting ULK1 through the activation of PI3K/AKT/mTOR signaling. These findings may provide a molecular basis for the application of BMSCs in therapies for patients with CLF. Additionally, identification of the regulatory role of PI3K/AKT/mTOR/ULK1 axis in HSCs activation provides potential targets for inhibiting HSCs activation-associated therapeutic strategies.

## Data Availability

The authors confirm that the data supporting the findings of this study are available within the article.
